# Feedback Schemes for the Action-Dependent Wiretap Channel with Noncausal State at the Transmitter

**DOI:** 10.3390/e21030278

**Published:** 2019-03-13

**Authors:** Haonan Zhang, Linman Yu, Bin Dai

**Affiliations:** 1 School of Information Science and Technology, Southwest Jiaotong University, Chengdu 611756, China; 2 School of Economics and Management, Chengdu Textile College, Chengdu 611731, China

**Keywords:** action-dependent channel, dirty paper channel, noiseless feedback, secrecy capacity, wiretap channel

## Abstract

In this paper, we propose two feedback coding schemes for the action-dependent wiretap channel with noncausal state at the transmitter. The first scheme follows from the already existing secret key based feedback coding scheme for the wiretap channel. The second one follows from our recently proposed hybrid feedback scheme for the wiretap channel. We show that, for the action-dependent wiretap channel with noncausal state at the transmitter, the second feedback scheme performs better than the first one, and the capacity results of this paper are further explained via a Gaussian example, which we call the action-dependent dirty paper wiretap channel with noiseless feedback.

## 1. Introduction

Using channel feedback to enhance the physical layer security (PLS) of a communication system was first proposed by Ahlswede and Cai [1], who re-visited the foundation of the PLS—the wiretap channel model [2]—by considering a noiseless feedback channel from the legitimate receiver to the transmitter. Ahlswede et al. [1] showed that, since the eavesdropper does not know the feedback, the legitimate receiver’s feedbackcan be used to generate secret keys shared between the transmitter and the legitimate receiver, and these keys can be used to encrypt the transmitted message. Using the feedback scheme in [1], it has been shown that the secrecy capacity (channel capacity with perfect secrecy constraint) of the wiretap channel can be enhanced. Furthermore, Ahlswede et al. [1] showed that this usage of feedback is optimal (achieving the secrecy capacity of the wiretap channel with noiseless feedback) if the channel is physically degraded (the eavesdropper’s received signal is a degraded version of the legitimate receiver’s). In recognition of this, Ardestanizadeh et al. [3] further pointed out that, if the noiseless feedback channel can be used to transmit anything the legitimate parties wish, the best choice of the legitimate parties is to send pure random bits (secret key) over the feedback channel. Subsequently, Schaefer et al. [4] extended the work of [3] to a broadcast situation, where two legitimate receivers of the broadcast channel independently send their secret keys to the transmitter via two noiseless feedback channels, and these keys help to enhance the achievable secrecy rate region of the broadcast wiretap channel [5]. Other related works in the PLS of the feedback channels include those by [6,7,8], who introduced channel state information (CSI) into various feedback channel models. Recently, Dai et al. [9] showed that, for the general wiretap channel (without physically degraded assumption), a better usage of the feedback is to generate not only key but also cooperative message from it, and this cooperative message helps the legitimate receiver to improve his decoding performance. Dai et al. [9] showed that this new feedback scheme achieves a larger achievable secrecy rate than the already existing secret key based feedback scheme [1] does.

Channel with noncausal state at the transmitter was first investigated by [10], and the capacity of this channel model was found by [11]. Subsequently, Costa et al. [12] studied the Gaussian case in [11], which is known as the dirty paper channel, and showed that the capacity of the dirty paper channel equals the capacity of the Gaussian channel without the state (also called interference). Here, note that the channel state in [10,11,12] is assumed to be independent of the transmitted message. In [13], the channel with noncausal or causal state available at the transmitter is revisited by considering the case that the transmitter can take actions on the channel state, i.e., the state is no longer independent of the transmitted message. This model is known as the action-dependent channel with states, and the capacity of this model is determined for both the noncausal and causal cases. Moreover, for the Gaussian case of the action-dependent channel with states (also called action-dependent dirty paper channel), it is shown that the actions on the state enhance the capacity of the dirty paper channel. Recently, a natural extension of the channel with noncausal state at the transmitter to the secrecy communication setting receives a lot of attention.Specifically, the authors of [14,15,16] studied the discrete memoryless wiretap channel with noncausal state at the transmitter, and proposed lower and upper bounds on its secrecy capacity. Mitrpant et al. [17] studied the Gaussian case in [14] (also called the dirty paper wiretap channel), and showed that the state (interference) non-causally known by the transmitter helps to enhance the secrecy capacity of the Gaussian wiretap channel [18]. Dai et al. [19] extended the state-dependent wiretap channel [14] to a broadcast situation, and proposed inner and outer bounds on the secrecy capacity region of this model. Dai et al. [20] studied the physically degraded action-dependent wiretap channel with noncausal state, and proposed lower and upper bounds on its secrecy capacity. Here, note that the action encoder in [20] is assumed to be deterministic, which implies that the output of the action encoder is a deterministic function of the transmitted message, and this leads to additional information leakage to the eavesdropper. Based on the work of [20,21] studied the feedback effect on the model proposed in [20]. A secret key based feedback scheme is provided in [21], and it is shown to be optimal for the physically degraded case.

**Figure 1:**
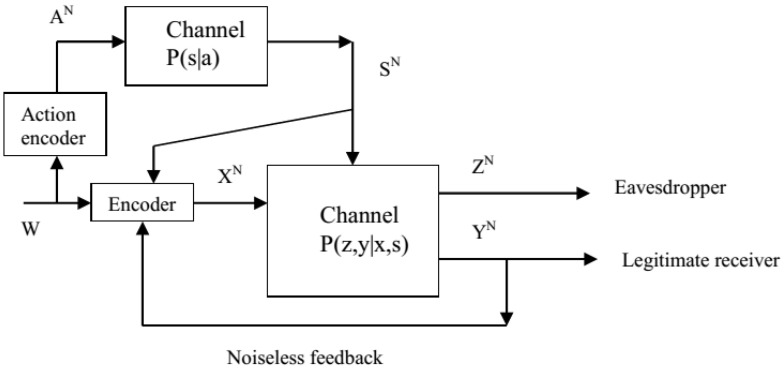
The action-dependent wiretap channel with noncausal state and noiseless feedback.

In this paper, we study the action-dependent wiretap channel with noncausal state and noiseless feedback (the model of this paper can be viewed as the model of [21] without physically degraded assumption and with stochastic action encoder) (see Figure 1), and try to answer the following two fundamental questions:

(1) How should the feedback scheme in [9] be extended to the action-dependent wiretap channel with noncausal state?
(2) For the action-dependent wiretap channel with noncausal state, does the hybrid feedback scheme in [9] still gain advantages over the traditional one used in [1,2,3,4,5,6,7,8]?

The main contribution of this paper includes:

(1) We propose a new lower bound on the secrecy capacity of the action-dependent wiretap channel with noncausal state and noiseless feedback, which is constructed according to a hybrid feedback scheme similar to that in [9].
(2) From a Gaussian example, which is also called the action-dependent dirty paper wiretap channel with noiseless feedback, we show that our new lower bound on the secrecy capacity is larger than the secret key based lower bound. Moreover, we find that our new lower bound achieves the secrecy capacity for some special cases.

The remainder of this paper is organized as follows. Section 2 is about the problem formulation and the main result of this paper. The achievability proof of our new lower bound on the secrecy capacity of the action-dependent wiretap channel with noncausal state and noiseless feedback is provided in Section 3. A Gaussian example and numerical results are provided in Section 4. Final conclusions are presented in Section 5.

## 2. Problem Formulation and New Result

*Notations:* For the rest of this manuscript, the random variables (RVs), values and alphabets are written in uppercase letters, lowercase letters and calligraphic letters, respectively. The random vectors and their values are denoted by a similar convention. For example, *Y* represents a RV, and *y* represents a value in the alphabet Y. Similarly, $Y^{N}$ represents a random *N*-vector $\left(Y_{1},...,Y_{N}\right)$, and $y^{N}=\left(y_{1},...,y_{N}\right)$ represents a vector value in $Y^{N}$ (the *N*th Cartesian power of Y). In addition, for an event X=x, its probability is denoted by P(x). In the remainder of this manuscript, the base of the log function is 2.

*Model description:* In Figure 1, the channel is discrete memoryless, i.e., the overall channel transition probability is given by

(1) $$P\left(y^{N},z^{N}|x^{N},s^{N}\right)=\prod_{i=1}^{N}P\left(z_{i},y_{i}|x_{i},s_{i}\right),$$

where $s_{i}\in S$, $x_{i}\in X$, $y_{i}\in Y$ and $z_{i}\in Z$. The message *W* is uniformly distributed in its alphabet W={1,2,...,|W|}, and a stochastic action encoder encodes *W* into an action sequence $A^{N}$. The channel state sequence $S^{N}$ is generated through a discrete memoryless channel (DMC) $A^{N}\to S^{N}$ with transition probability P(s|a). Since $S^{N}$ is non-causally known by the channel encoder and the legitimate receiver’s channel output is sent back to the transmitter, the *i*th (i∈{1,2,...,N}) channel input $X_{i}=f_{i}\left(W,S^{N},Y^{i-1}\right)$, where $f_{i}$ is a stochastic encoding function. The legitimate receiver produces an estimation $\hat{W}=\psi \left(Y^{N}\right)$ (ψ is the legitimate receiver’s decoding function), and the average decoding error probability equals

(2) $$P_{e}=\frac{1}{\left|W\right|}\sum_{i\in W}Pr\left\{\psi \left(y^{N}\right)\neq i|i\;\;\mathrm{sent}\right\}.$$

The eavesdropper’s equivocation rate of the message *W* is formulated as

(3) $$\Delta =\frac{1}{N}H\left(W|Z^{N}\right).$$

Given a positive number *R*, if for arbitrarily small ϵ and sufficiently large *N*, there exist a pair of channel encoder and decoder described above such that

(4) $$\begin{array}{cc} & \frac{\log|W|}{N}\geq R-\epsilon ,\;\;\Delta \geq R-\epsilon ,\;\;P_{e}\leq \epsilon , \end{array}$$

we say *R* is achievable with weak perfect secrecy. The secrecy capacity $C_{sa}^{f}$ consists of all achievable weak secrecy rates, and bounds on $C_{sa}^{f}$ are given in the following theorems and corollary.

**Theorem** **1.** $C_{sa}^{f}\geq R_{sa}^{f*}$, where

(5) $$\begin{array}{c} R_{sa}^{f*}=\max\min\left\{I\left(U;Y\right)-I\left(U;S|A\right),{\left[I\left(U;V,Y\right)-I\left(U;Z\right)\right]}^{+}+H\left(Y|U,Z\right)\right\}, \end{array}$$

*${\left[x\right]}^{+}=x$ for x≥0, else ${\left[x\right]}^{+}=0$, and the joint distribution is denoted by*

(6) $$\begin{array}{c} P(u,v,a,s,x,y,z)=P(v|u,y)P(y,z|x,s)P(x|u,s)P(u|a,s)P(a,s). \end{array}$$

**Proof.**  The lower bound $R_{sa}^{f*}$ is achieved by combining the binning scheme in [13] with the hybrid coding scheme in [9], and the details about the proof are in Section 3. ☐

The following lower bound $R_{sa}^{f**}$ in Corollary 1 can be directly obtained from Theorem 1 by letting *V* be constant, and this lower bound can be viewed as a secret key based lower bound (application of the secret key based feedback strategy [1] to the model of Figure 1) on $C_{sa}^{f}$.

**Corollary** **1.** $C_{sa}^{f}\geq R_{sa}^{f**}$, where

(7) $$\begin{array}{c} R_{sa}^{f**}=\max\min\left\{I\left(U;Y\right)-I\left(U;S|A\right),{\left[I\left(U;Y\right)-I\left(U;Z\right)\right]}^{+}+H\left(Y|U,Z\right)\right\}, \end{array}$$

*and the joint distribution is denoted by*

(8) $$\begin{array}{c} P(u,a,s,x,y,z)=P(y,z|x,s)P(x|u,s)P(u|a,s)P(a,s). \end{array}$$

**Remark** **1.** Note that [21] also proposed a secret key based lower bound on the secrecy capacity of the physically degraded action-dependent wiretap channel with noncausal state and noiseless feedback. However, we should point out that the model studied in [21] assumes the action encoder is a deterministic encoder, i.e., if the eavesdropper knows $A^{N}$, he also knows the message W. Hence, our lower bound $R_{sa}^{f**}$ generalizes that in [21] as the deterministic action encoder is a special case of the stochastic one studied in this paper and there is no physically degraded assumption in this paper.

Besides the above lower bounds on $C_{sa}^{f}$, the following theorem shows a simple upper bound on $C_{sa}^{f}$.

**Theorem** **2.** *$C_{sa}^{f}\leq C_{sa}^{f-out}$, where*

(9) $$\begin{array}{c} C_{sa}^{f-out}=\max\left(I\left(U;Y\right)-I\left(U;S|A\right)\right), \end{array}$$

*and the joint distribution is denoted by Equation (8).*

**Proof.**  Since $C_{sa}^{f}$ cannot exceed the capacity of the model in Figure 1 without eavesdropper, we know that $C_{sa}^{f}$ is upper bounded by the capacity of the action-dependent channel with feedback. In [13], it has been shown that feedback does not increase the capacity of the action-dependent channel (max(I(U;Y)-I(U;S|A))), hence Theorem 2 is proved. The proof of Theorem 2 is completed. ☐

In Section 4, the above proposed hybrid lower bound $R_{sa}^{f*}$ ise compared with the secret key based lower bound $R_{sa}^{f**}$ via a Gaussian example, and we show which feedback strategy performs better.

## 3. Proof of Theorem 1

In this section, the hybrid feedback strategy for the wiretap channel [9] and the binning scheme for the action-dependent channel with noncausal state at the transmitter [13] are combined to show the achievability of Theorem 1. The rest of this section is organized as follows. The code-book construction and the transmission scheme are described in Section 3.1, and the equivocation analysis of the proposed scheme is shown in Section 3.2.

### 3.1. Code-Book Construction and Transmission Scheme

*Definitions and notations*:

- Similar to the coding scheme in [9], suppose that the overall transmission consists of *B* blocks, and the codeword length in each block is *N*.
- The overall message *W* is composed of *B* components ($W=(W_{1},...,W_{B})$), and each component $W_{b}$ (b∈{1,2,...,B}) is the message transmitted in block *b*. The value of $W_{b}$ belongs to the set $\left\{1,...,2^{NR}\right\}$. Next, split $W_{b}$ into two parts $W_{b}=\left(W_{b,1},W_{b,2}\right)$, and the values of $W_{b,1}$ and $W_{b,2}$, respectively, belong to the sets $\left\{1,...,2^{NR_{1}}\right\}$ and $\left\{1,...,2^{NR_{2}}\right\}$. Note that $R_{1}+R_{2}=R$.
- Analogously, the randomly produced dummy messages $W^{{}^{\prime }}$ and $W^{{}^{\prime \prime }}$, which are used to confuse the wiretapper, also consist of *B* components ($W^{{}^{\prime }}=\left(W_{1}^{{}^{\prime }},...,W_{B}^{{}^{\prime }}\right)$ and $W^{{}^{\prime \prime }}=\left(W_{1}^{{}^{\prime \prime }},...,W_{B}^{{}^{\prime \prime }}\right)$), and the components $W_{b}^{{}^{\prime }}$ and $W_{b}^{{}^{\prime \prime }}$ (b∈{1,2,...,B}) are transmitted in block *b*. Here, note that $W_{b}^{{}^{\prime }}$ and $W_{b}^{{}^{\prime \prime }}$ are uniformly drawn from the sets $\left\{1,...,2^{NR^{{}^{\prime }}}\right\}$ and $\left\{1,...,2^{NR^{{}^{\prime \prime }}}\right\}$, respectively.
- The auxiliary message $W^{*}$, which is used to cooperate with the channel state, consists of *B* components ($W^{*}=\left(W_{1}^{*},...,W_{B}^{*}\right)$), and the value of $W_{b}^{*}$ (b∈{1,2,...,B}) belongs to the set $\left\{1,...,2^{NR^{*}}\right\}$.
- The help information $W^{**}$ and $W^{***}$, which is used to ameliorate the legitimate receiver’s decoding performance, consists of *B* components ($W^{**}=\left(W_{1}^{**},...,W_{B}^{**}\right)$ and $W^{***}=\left(W_{1}^{***},...,W_{B}^{***}\right)$), and the value of $W_{b}^{**}$ and $W_{b}^{***}$ (b∈{1,2,...,B}), respectively, belongs to the sets $\left\{1,...,2^{NR^{**}}\right\}$ and $\left\{1,...,2^{NR^{***}}\right\}$.
- In block *b* (1≤b≤B), the random vectors $A^{N}$, $X^{N}$, $Y^{N}$, $Z^{N}$, $S^{N}$, $U^{N}$ and $V^{N}$ are denoted by ${\bar{A}}_{b}$, ${\bar{X}}_{b}$, ${\bar{Y}}_{b}$, ${\bar{Z}}_{b}$, ${\bar{S}}_{b}$, ${\bar{U}}_{b}$ and ${\bar{V}}_{b}$, respectively. In addition, let $X^{B}=\left({\bar{X}}_{1},...,{\bar{X}}_{B}\right)$ be a collection of the random vectors $X^{N}$ for all blocks. Analogously, we have $A^{B}=\left({\bar{A}}_{1},...,{\bar{A}}_{B}\right)$, $Y^{B}=\left({\bar{Y}}_{1},...,{\bar{Y}}_{B}\right)$, $Z^{B}=\left({\bar{Z}}_{1},...,{\bar{Z}}_{B}\right)$, $S^{B}=\left({\bar{S}}_{1},...,{\bar{S}}_{B}\right)$, $U^{B}=\left({\bar{U}}_{1},...,{\bar{U}}_{B}\right)$ and $V^{B}=\left({\bar{V}}_{1},...,{\bar{V}}_{B}\right)$. The vector value is written in lower case letter.

*Code-book generation*:

- In block *b* (1≤b≤B), randomly produce $2^{N(R_{1}+R_{2}+R^{{}^{\prime \prime }})}$ i.i.d. codewords ${\bar{a}}_{b}$ with respect to (w.r.t.) P(a), and label them as ${\bar{a}}_{b}\left(w_{b,1},w_{b,2},w_{b}^{{}^{\prime \prime }}\right)$, where $w_{b,1}\in \left\{1,2,...,2^{NR_{1}}\right\}$, $w_{b,2}\in \left\{1,2,...,2^{NR_{2}}\right\}$ and $w_{b}^{{}^{\prime \prime }}\in \left\{1,2,...,2^{NR^{{}^{\prime \prime }}}\right\}$.
- In block *b* (1≤b≤B), randomly produce $2^{N(R_{1}+R_{2}+R^{{}^{\prime }}+R^{*}+R^{**})}$ i.i.d. codewords ${\bar{u}}_{b}$ w.r.t. P(u|a,s), and label them as ${\bar{u}}_{b}\left(w_{b,1},w_{b,2},w_{b}^{{}^{\prime }},w_{b}^{*},w_{b-1}^{**}\right)$, where $w_{b,1}\in \left\{1,2,...,2^{NR_{1}}\right\}$, $w_{b,2}\in \left\{1,2,...,2^{NR_{2}}\right\}$, $w_{b}^{{}^{\prime }}\in \left\{1,2,...,2^{NR^{{}^{\prime }}}\right\}$, $w_{b}^{*}\in \left\{1,2,...,2^{NR^{*}}\right\}$ and $w_{b-1}^{**}\in \left\{1,2,...,2^{NR^{**}}\right\}$.
- For each possible value of ${\bar{u}}_{b}\left(w_{b,1},w_{b,2},w_{b}^{{}^{\prime }},w_{b}^{*},w_{b-1}^{**}\right)$ and ${\bar{y}}_{b}$, randomly produce $2^{N(R^{**}+R^{***})}$ i.i.d. codewords ${\bar{v}}_{b}$ on the basis of P(v|u,y). Then, label these ${\bar{v}}_{b}$ as ${\bar{v}}_{b}\left(w_{b}^{**},w_{b}^{***}\right)$, where $w_{b}^{**}\in \left\{1,2,...,2^{NR^{**}}\right\}$ and $w_{b}^{***}\in \left\{1,2,...,2^{NR^{***}}\right\}$.
- For given ${\bar{u}}_{b}$ and ${\bar{s}}_{b}$, the transmitted sequence ${\bar{x}}_{b}$ is i.i.d. produced on the basis of the probability P(x|u,s).

*Encoding scheme*:

- For block 1, the transmitter chooses ${\bar{a}}_{1}\left(w_{1,1},w_{1,2}=1,w_{1}^{{}^{\prime \prime }}\right)$. Next, define $w_{0}^{**}=1$, for given ${\bar{a}}_{1}\left(w_{1,1},w_{1,2}=1,w_{1}^{{}^{\prime \prime }}\right)$ and the state sequence ${\bar{s}}_{1}$, the transmitter selects an index $w_{1}^{*}$ such that $\left({\bar{u}}_{1}\left(w_{1,1},w_{1,2}=1,w_{1}^{{}^{\prime }},w_{1}^{*},w_{0}^{**}=1\right),{\bar{a}}_{1}\left(w_{1,1},w_{1,2}=1,w_{1}^{{}^{\prime \prime }}\right),{\bar{s}}_{1}\right)$ are jointly typical. If no such $w_{1}^{*}$ exists, declare an encoding error. If multiple $w_{1}^{*}$ exist, randomly pick out one. Based on the Covering Lemma [22], the encoding error tends to zero if
  (10) $$\begin{array}{cc} & R^{*}>I\left(U;S|A\right). \end{array}$$
- For block *b* (i∈{2,3,...,B-1}), before transmission, produce a mapping $g_{b}:{\bar{y}}_{b-1}\to \left\{1,2,...,2^{NR_{2}}\right\}$ (this mapping is generated exactly the same as that in [1]). Based on this mapping, generate a random variable (RV) $K_{b}=g_{b}\left({\bar{Y}}_{b-1}\right)$ taking values in $\left\{1,2,...,2^{NR_{2}}\right\}$, and $Pr\left\{K_{b}=j\right\}=2^{-NR_{2}}$ for $j\in \{1,2,...,2^{NR_{2}}\}$. The RV $K_{b}$ is used as a secret key and it is not known to the eavesdropper, and $K_{b}$ is independent of the real transmitted messages $W_{b,1}$ and $W_{b,2}$ for block *b*. Notice that $k_{b}=g_{b}\left({\bar{y}}_{b-1}\right)\in \left\{1,2,...,2^{NR_{2}}\right\}$ is a realization of $K_{b}$. The mapping $g_{b}$ is revealed to all parties. First, since the transmitter knows its own ${\bar{u}}_{b-1}\left(w_{b-1,1},w_{b-1,2}⊕k_{b-1},w_{b-1}^{{}^{\prime }},w_{b-1}^{*},w_{b-2}^{**}\right)$, ${\bar{a}}_{b-1}\left(w_{b-1,1},w_{b-1,2},w_{b-1}^{{}^{\prime \prime }}\right)$, ${\bar{s}}_{b-1}$ and ${\bar{y}}_{b-1}$, he tries to find a ${\bar{v}}_{b-1}\left(w_{b-1}^{**},w_{b-1}^{***}\right)$ such that $\left({\bar{v}}_{b-1},{\bar{u}}_{b-1},{\bar{y}}_{b-1},{\bar{s}}_{b-1},{\bar{a}}_{b-1}\right)$ are jointly typical. For the case that more than one ${\bar{v}}_{b-1}$ exist, randomly pick one; if no such ${\bar{v}}_{b-1}$ exists, declare an encoding error. According to the Covering Lemma [22], the encoding error approaches to zero if
  (11) $$\begin{array}{cc} & R^{**}+R^{***}\geq I\left(V;U,Y,A,S\right)\overset{\left(1\right)}{=}I\left(V;U,Y\right), \end{array}$$
  where (1) is from the definition in Equation (6), which implies that V→(U,Y)→(A,S). Next, the transmitter chooses ${\bar{a}}_{b}\left(w_{b,1},w_{b,2},w_{b}^{{}^{\prime \prime }}\right)$. Finally, since the transmitter obtains ${\bar{v}}_{b-1}\left(w_{b-1}^{**},w_{b-1}^{***}\right)$, he extracts $w_{b-1}^{**}$ and tries to find a $w_{b}^{*}$ such that $\left({\bar{u}}_{b}\left(w_{b,1},w_{b,2}⊕k_{b},w_{b}^{{}^{\prime }},w_{b}^{*},w_{b-1}^{**}\right),{\bar{a}}_{b}\left(w_{b,1},w_{b,2},w_{b}^{{}^{\prime \prime }}\right),{\bar{s}}_{b}\right)$ are jointly typical. If no such $w_{b}^{*}$ exists, declare an encoding error. If multiple $w_{b}^{*}$ exist, randomly pick out one. Based on the Covering Lemma [22], the encoding error tends to zero if Equation (10) holds. The codeword ${\bar{u}}_{b}\left(w_{b,1},w_{b,2}⊕k_{b},w_{b}^{{}^{\prime }},w_{b}^{*},w_{b-1}^{**}\right)$ is picked for transmission.
- At block *B*, first, the transmitter chooses ${\bar{a}}_{B}\left(1,1,1\right)$. Next, after receiving the feedback ${\bar{y}}_{B-1}$, the transmitter tries to find a ${\bar{v}}_{B-1}\left(w_{B-1}^{**},w_{B-1}^{***}\right)$ such that $\left({\bar{v}}_{B-1}\left(w_{B-1}^{**},w_{B-1}^{***}\right),{\bar{u}}_{B-1},{\bar{y}}_{B-1},{\bar{s}}_{B-1},{\bar{a}}_{B-1}\right)$ are jointly typical. After decoding such ${\bar{v}}_{B-1}\left(w_{B-1}^{**},w_{B-1}^{***}\right)$, the transmitter extracts $w_{B-1}^{**}$ and tries to find a $w_{B}^{*}$ such that $\left({\bar{u}}_{B}\left(1,1,1,w_{B}^{*},w_{B-1}^{**}\right),{\bar{a}}_{B}\left(1,1,1\right),{\bar{s}}_{B}\right)$ are jointly typical. If no such $w_{B}^{*}$ exists, declare an encoding error. If multiple $w_{B}^{*}$ exist, randomly pick out one. The codeword ${\bar{u}}_{B}\left(1,1,1,w_{B}^{*},w_{B-1}^{**}\right)$ is picked for transmission.

*Decoding scheme*: 

The decoding procedure starts from block *B*. At block *B*, the legitimate receiver chooses a ${\bar{u}}_{B}\left(1,1,1,w_{B}^{*},w_{B-1}^{**}\right)$ which is jointly typical with ${\bar{y}}_{B}$ and ${\bar{a}}_{B}\left(1,1,1\right)$. For the case that more than one or no such ${\bar{u}}_{B}$ exists, declare a decoding error. Based on the Packing Lemma [22] and a similar argument in [13], this kind of decoding error approaches to zero when

(12) $$\begin{array}{c} R^{*}+R^{**}\leq I\left(U;Y\right). \end{array}$$

After decoding ${\bar{u}}_{B}$, the legitimate receiver extracts $w_{B-1}^{**}$ from it. Then, he tries to select only one ${\bar{v}}_{B-1}\left(w_{B-1}^{**},w_{B-1}^{***}\right)$ such that given $w_{B-1}^{**}$, ${\bar{v}}_{B-1}$ is jointly typical with ${\bar{y}}_{B-1}$. For the case that more than one or no such ${\bar{v}}_{B-1}$ exist, declare a decoding error. Based on the Packing Lemma [22], this kind of decoding error approaches to zero when

(13) $$\begin{array}{c} R^{***}\leq I\left(V;Y\right). \end{array}$$

After obtaining such unique ${\bar{v}}_{B-1}$, the legitimate receiver tries to find only one pair of $\left({\bar{u}}_{B-1},{\bar{a}}_{B-1}\right)$ such that $\left({\bar{y}}_{B-1},{\bar{a}}_{B-1},{\bar{v}}_{B-1},{\bar{u}}_{B-1}\right)$ are jointly typical. Based on the Packing Lemma [22] and a similar argument in [13], this kind of decoding error approaches to zero when

(14) $$\begin{array}{c} R_{1}+R_{2}+R^{{}^{\prime }}+R^{*}+R^{**}+R^{{}^{\prime \prime }}\leq I\left(U;V,Y\right). \end{array}$$

**Figure 2:**
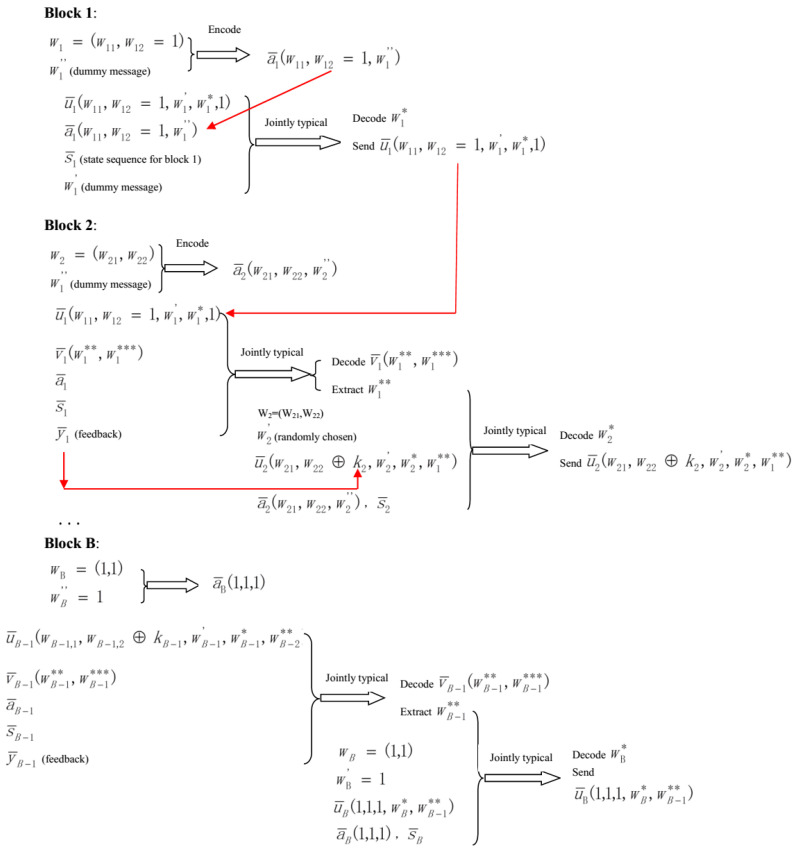
The encoding procedure for all blocks.

**Figure 3:**
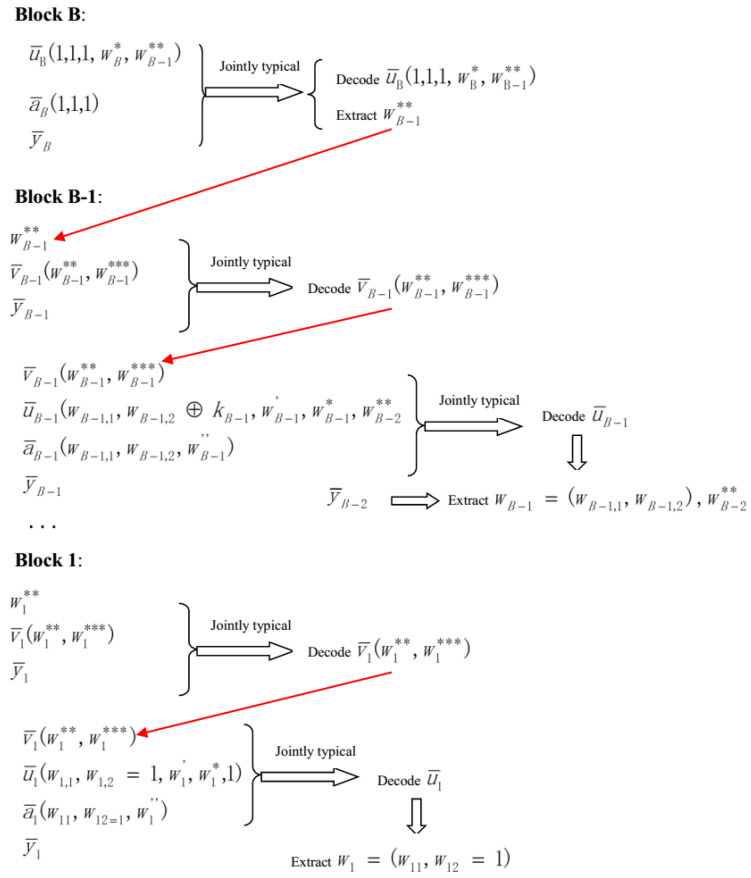
The decoding procedure for all blocks.

After decoding ${\bar{u}}_{B-1}$, the legitimate receiver picks out $w_{B-1,1}$, $w_{B-1,2}⊕k_{B-1}$, $w_{B-2}^{**}$ from it. Note that the legitimate receiver has full knowledge of $k_{B-1}=g_{B-1}\left({\bar{y}}_{B-2}\right)$, and hence he obtains the message $w_{B-1}=\left(w_{B-1,1},w_{B-1,2}\right)$. Analogously, the legitimate receiver decodes the messages $w_{B-2},w_{B-3},...,w_{1}$, and the decoding procedure is completed. For convenience, the encoding and decoding schemes are explained by the following Figure 2 and Figure 3, respectively.

### 3.2. Equivocation Analysis

The overall equivocation Δ, which is denoted by $\Delta =\frac{1}{BN}H\left(W|Z^{B}\right)$, is given by

(15) $$\begin{array}{c} \Delta \overset{\left(a\right)}{=}\frac{1}{BN}\left(H\left({\tilde{W}}_{1}|Z^{B}\right)+H\left({\tilde{W}}_{2}|Z^{B},{\tilde{W}}_{1}\right)\right), \end{array}$$

where (a) is due to the definitions ${\tilde{W}}_{1}=\left(W_{1,1},...,W_{B,1}\right)$ and ${\tilde{W}}_{2}=\left(W_{1,2},...,W_{B,2}\right)$.

The term $H({\tilde{W}}_{1}|Z^{B})$ in Equation (15) can be bounded by

(16) $$\begin{array}{c} H\left({\tilde{W}}_{1}|Z^{B}\right)=H\left({\tilde{W}}_{1},Z^{B}\right)-H\left(Z^{B}\right)\\ =H\left({\tilde{W}}_{1},Z^{B},U^{B}\right)-H\left(U^{B}|{\tilde{W}}_{1},Z^{B}\right)-H\left(Z^{B}\right)\\ \overset{\left(b\right)}{=}H\left(U^{B}\right)-H\left(U^{B}|{\tilde{W}}_{1},Z^{B}\right)-I\left(U^{B};Z^{B}\right)\\ \overset{\left(c\right)}{=}\left(B-1\right)NR_{1}+\left(B-2\right)NR_{2}+\left(B-1\right)NR^{{}^{\prime }}+BNR^{*}+\left(B-1\right)NR^{**}-BNI\left(U;Z\right)-H\left(U^{B}|{\tilde{W}}_{1},Z^{B}\right)\\ \overset{\left(d\right)}{\geq }\left(B-1\right)NR_{1}+\left(B-2\right)NR_{2}+\left(B-1\right)NR^{{}^{\prime }}+BNR^{*}+\left(B-1\right)NR^{**}-BNI\left(U;Z\right)-BN\epsilon_{3}, \end{array}$$

where (b) is implied by $H({\tilde{W}}_{1}|U^{B})=0$, (c) is due to the construction of $U^{B}$ and the channel is memoryless, and (d) is due to that given ${\tilde{w}}_{1}$ and $z^{B}$, the eavesdropper attempts to find a unique $u^{B}$ that is jointly typical with his own received signals $z^{B}$, and according to the Packing Lemma [22], we can conclude that the eavesdropper’s decoding error tends to zero if

(17) $$\begin{array}{c} R_{2}+R^{{}^{\prime }}+R^{*}+R^{**}\leq I\left(U;Z\right), \end{array}$$

then applying Fano’s inequality, $\frac{1}{BN}H\left(U^{B}|{\tilde{W}}_{1},Z^{B}\right)\leq \epsilon_{3}$ is obtained, where $\epsilon_{3}\to 0$ while B,N→∞.

Moreover, the term $H({\tilde{W}}_{2}|Z^{B},{\tilde{W}}_{1})$ in Equation (15) can be bounded by

(18) $$\begin{array}{c} H({\tilde{W}}_{2}|Z^{B},{\tilde{W}}_{1})\\ \geq \sum_{i=2}^{B-1}H\left(W_{i,2}|Z^{B},{\tilde{W}}_{1},W_{1,2}=1,...,W_{i-1,2},W_{i,2}⊕K_{i}\right)\\ \overset{\left(e\right)}{=}\sum_{i=2}^{B-1}H\left(W_{i,2}|{\bar{Z}}_{i-1},W_{i,2}⊕K_{i}\right)\\ \geq \sum_{i=2}^{B-1}H\left(W_{i,2}|{\bar{Z}}_{i-1},{\bar{U}}_{i-1},W_{i,2}⊕K_{i}\right)\\ =\sum_{i=2}^{B-1}H\left(K_{i}|{\bar{Z}}_{i-1},{\bar{U}}_{i-1},W_{i,2}⊕K_{i}\right)\\ \overset{\left(f\right)}{=}\sum_{i=2}^{B-1}H\left(K_{i}|{\bar{Z}}_{i-1},{\bar{U}}_{i-1}\right)\\ \overset{\left(g\right)}{\geq }\left(B-2\right)\left(\log\frac{1-\epsilon_{1}}{1+\delta }+N\left(1-\epsilon_{2}\right)H\left(Y|U,Z\right)\right), \end{array}$$

where (e) is due to the Markov chain $W_{i,2}\to \left({\bar{Z}}_{i-1},W_{i,2}⊕K_{i}\right)\to \left({\tilde{W}}_{1},W_{1,2},...,W_{i-1,2},{\bar{Z}}_{1},...,{\bar{Z}}_{i-2},{\bar{Z}}_{i},...,{\bar{Z}}_{B}\right)$, (f) follows by $K_{i}\to \left({\bar{Z}}_{i-1},{\bar{U}}_{i-1}\right)\to W_{i,2}⊕K_{i}$, and (g) is from the balanced coloring Lemma [9] (p. 264), i.e., given ${\bar{z}}_{i-1}$ and ${\bar{u}}_{i-1}$, there are at least $\frac{\gamma }{1+\delta }$ colors, which implies that

(19) $$\begin{array}{c} H\left(K_{i}|{\bar{Z}}_{i-1},{\bar{U}}_{i-1}\right)\geq \log\frac{1-\epsilon_{1}}{1+\delta }+N\left(1-\epsilon_{2}\right)H\left(Y|U,Z\right), \end{array}$$

where $\epsilon_{1}$, $\epsilon_{2}$ and δ approach to 0 as *N* goes to infinity.

Substituting Equations (16) and (18) into Equation (15), we have

(20) $$\begin{array}{c} \Delta \geq R^{*}+\frac{B-1}{B}\left(R_{1}+R^{{}^{\prime }}+R^{**}\right)+\frac{B-2}{B}R_{2}-I\left(U;Z\right)-\epsilon_{3}\\ +\frac{B-2}{BN}\log\frac{1-\epsilon_{1}}{1+\delta }+\frac{B-2}{B}\left(1-\epsilon_{2}\right)H\left(Y|U,Z\right). \end{array}$$

The bound in Equation (20) indicates that if

(21) $$\begin{array}{c} R^{{}^{\prime }}+R^{*}+R^{**}\geq I\left(U;Z\right)-H\left(Y|U,Z\right), \end{array}$$

$\Delta \geq R_{1}+R_{2}-\epsilon$ can be proved by choosing sufficiently large *B* and *N*.

Now combining Equation (11) with Equation (13), we have

(22) $$\begin{array}{c} R^{**}\geq I\left(U,Y;V\right)-I\left(Y;V\right)=I\left(V;U|Y\right). \end{array}$$

Next, from Equations (22), (10) and (14), we can conclude that

(23) $$\begin{array}{c} R_{1}+R_{2}+R^{{}^{\prime }}+R^{{}^{\prime \prime }}\leq I\left(Y,V;U\right)-I\left(V;U|Y\right)-I\left(U;S|A\right)=I\left(U;Y\right)-I\left(U;S|A\right). \end{array}$$

Then, implied by Equations (21) and (14), we have

(24) $$\begin{array}{c} R_{1}+R_{2}+R^{{}^{\prime \prime }}\leq I\left(Y,V;U\right)-I\left(U;Z\right)+H\left(Y|U,Z\right). \end{array}$$

Finally, applying Fourier–Motzkin elimination to remove $R_{1}$, $R_{2}$ ($R=R_{1}+R_{2}$), $R^{{}^{\prime }}$, $R^{{}^{\prime \prime }}$, $R^{*}$ and $R^{**}$ from Equations (22), (23), (24), (12), (14), (17) and (21), Theorem 1 is proved.

## 4. The Action-Dependent Dirty Paper Wiretap Channel with Noiseless Feedback

**Figure 4:**
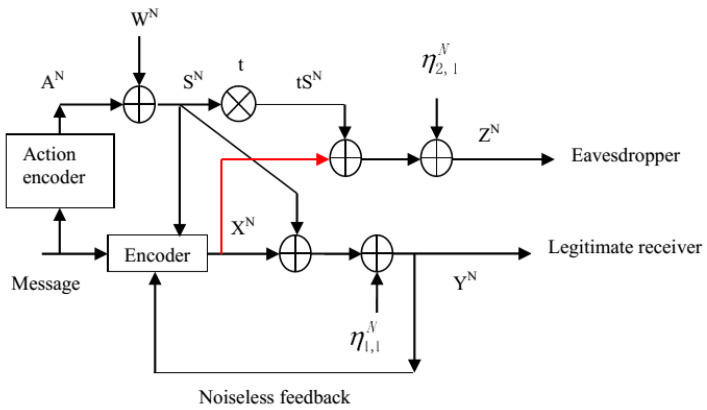
The action-dependent dirty paper wiretap channel with noiseless feedback.

The Gaussian case of the action-dependent wiretap channel with noncausal state at the transmitter and feedback, which we also call the action-dependent dirty paper wiretap channel with noiseless feedback, is depicted in Figure 4. At time *i* (i∈{1,2,...,N}), the inputs and outputs of this Gaussian model satisfy

(25) $$\begin{array}{cc} & S_{i}=A_{i}+W_{i},\;\;Y_{i}=X_{i}+S_{i}+\eta_{1,i},\;\;Z_{i}=X_{i}+tS_{i}+\eta_{2,i}, \end{array}$$

where $X_{i}$ is the channel input subject to an average power constraint *P*, $A_{i}$ is the output of the action encoder subject to an average power constraint $P_{A}$, *t* is a constant, and $W_{i}$, $\eta_{1,i}$, $\eta_{2,i}$ are independent Gaussian noises and are i.i.d. across the time index *i*. Here, note that $W_{i}∼N\left(0,\sigma_{w}^{2}\right)$, $\eta_{1,i}∼N\left(0,\sigma_{1}^{2}\right)$ and $\eta_{2,i}∼N\left(0,\sigma_{2}^{2}\right)$. The secrecy capacity of the action-dependent dirty paper wiretap channel with feedback is denoted by $C_{sag}^{f}$, and the lower and upper bounds on $C_{sag}^{f}$ will be given in the remainder of this section.

Before we show the bounds on $C_{sag}^{f}$, define

(26) $$\begin{array}{cc} & A∼N(0,P_{A}),\;\;X=\alpha A+\gamma W+G,\;\;U=\delta X+A+\beta W, \end{array}$$

where $\alpha^{2}P_{A}+\gamma^{2}\sigma_{w}^{2}\leq P$, $G∼N(0,P-\alpha^{2}P_{A}-\gamma^{2}\sigma_{w}^{2})$ and *G*, *A*, *W*, $\eta_{1}$, $\eta_{2}$ are independent of each other. Note that the definitions in Equation (26) are exactly the same as those in the action-dependent dirty paper channel [13]. Further, define

(27) $$\begin{array}{cc} & D=P-\alpha^{2}P_{A}-\gamma^{2}\sigma_{w}^{2}, \end{array}$$

(28) $$\begin{array}{cc} & E\left(U^{2}\right)={\left(1+\delta \alpha \right)}^{2}P_{A}+{\left(\delta \gamma +\beta \right)}^{2}\sigma_{w}^{2}+\delta^{2}D, \end{array}$$

(29) $$\begin{array}{cc} & E\left(Y^{2}\right)={\left(\alpha +1\right)}^{2}P_{A}+{\left(\gamma +1\right)}^{2}\sigma_{w}^{2}+D+\sigma_{1}^{2}, \end{array}$$

(30) $$\begin{array}{cc} & E\left(Z^{2}\right)={\left(\alpha +t\right)}^{2}P_{A}+{\left(\gamma +t\right)}^{2}\sigma_{w}^{2}+D+\sigma_{2}^{2}, \end{array}$$

(31) $$\begin{array}{cc} & E\left(UY\right)=\left(1+\delta \alpha \right)\left(1+\alpha \right)P_{A}+\left(\delta \gamma +\beta \right)\left(1+\gamma \right)\sigma_{w}^{2}+\delta D, \end{array}$$

(32) $$\begin{array}{cc} & E\left(UZ\right)=\left(1+\delta \alpha \right)\left(t+\alpha \right)P_{A}+\left(\delta \gamma +\beta \right)\left(t+\gamma \right)\sigma_{w}^{2}+\delta D, \end{array}$$

(33) $$\begin{array}{cc} & E\left(YZ\right)=\left(1+\alpha \right)\left(t+\alpha \right)P_{A}+\left(\gamma +1\right)\left(\gamma +t\right)\sigma_{w}^{2}+D, \end{array}$$

(34) $$\begin{array}{cc} & L=det\left(\begin{array}{ccc} E(U^{2}) & E(UY) & E(UZ)\\ E(UY) & E(Y^{2}) & E(YZ)\\ E(UZ) & E(YZ) & E(Z^{2}) \end{array}\right). \end{array}$$

First, substituting Equations (26) and (25) into Equation (5), our new lower bound $R_{sag}^{f*}$ on $C_{sag}^{f}$ is given by the following Theorem 3.

**Theorem** **3.** $C_{sag}^{f}\geq R_{sag}^{f*}$, where

(35) $$\begin{array}{c} R_{sag}^{f*}=\max_{\alpha ,\gamma ,\delta ,\beta }\min\{\frac{1}{2}\log\left(\frac{E\left(Y^{2}\right)E\left(U^{2}\right)}{E\left(Y^{2}\right)E\left(U^{2}\right)-{\left(E\left(UY\right)\right)}^{2}}\right)-\frac{1}{2}\log\left(\frac{{\left(\gamma \delta +\beta \right)}^{2}\sigma_{w}^{2}+\delta^{2}D}{\delta^{2}D}\right),\\ {\left[\frac{1}{2}\log\left(2\pi eE\left(U^{2}\right)\right)-\frac{1}{2}\log\left(\frac{E\left(Z^{2}\right)E\left(U^{2}\right)}{E\left(Z^{2}\right)E\left(U^{2}\right)-{\left(E\left(UZ\right)\right)}^{2}}\right)\right]}^{+}+\frac{1}{2}\log\left(\frac{2\pi eL}{E\left(Z^{2}\right)E\left(U^{2}\right)-{\left(E\left(UZ\right)\right)}^{2}}\right)\}, \end{array}$$

*and ${\left[x\right]}^{+}=x$ for x≥0, else ${\left[x\right]}^{+}=0$.*

Second, substituting Equations (26) and (25) into Equation (7), the secret key based lower bound $R_{sag}^{f**}$ on $C_{sag}^{f}$ is given by the following Theorem 4.

**Theorem** **4.** $C_{sag}^{f}\geq R_{sag}^{f**}$, where

(36) $$\begin{array}{c} R_{sag}^{f**}=\max_{\alpha ,\gamma ,\delta ,\beta }\min\{\frac{1}{2}\log\left(\frac{E\left(Y^{2}\right)E\left(U^{2}\right)}{E\left(Y^{2}\right)E\left(U^{2}\right)-{\left(E\left(UY\right)\right)}^{2}}\right)-\frac{1}{2}\log\left(\frac{{\left(\gamma \delta +\beta \right)}^{2}\sigma_{w}^{2}+\delta^{2}D}{\delta^{2}D}\right),\\ {\left[\frac{1}{2}\log\left(\frac{E\left(Y^{2}\right)E\left(U^{2}\right)}{E\left(Y^{2}\right)E\left(U^{2}\right)-{\left(E\left(UY\right)\right)}^{2}}\right)-\frac{1}{2}\log\left(\frac{E\left(Z^{2}\right)E\left(U^{2}\right)}{E\left(Z^{2}\right)E\left(U^{2}\right)-{\left(E\left(UZ\right)\right)}^{2}}\right)\right]}^{+}+\frac{1}{2}\log\left(\frac{2\pi eL}{E\left(Z^{2}\right)E\left(U^{2}\right)-{\left(E\left(UZ\right)\right)}^{2}}\right)\}, \end{array}$$

*and ${\left[x\right]}^{+}=x$ for x≥0, else ${\left[x\right]}^{+}=0$.*

Third, substituting Equations (26) and (25) into Equation (9), the upper bound $C_{sag}^{f-out}$ on $C_{sag}^{f}$ is given by Theorem 5.

**Theorem** **5.** *$C_{sag}^{f}\leq C_{sag}^{f-out}$, where*

(37) $$\begin{array}{c} C_{sag}^{f-out}=\frac{1}{2}\log\left(\max_{\left(\alpha ,\gamma \right):\alpha^{2}P_{A}+\gamma^{2}\sigma_{w}^{2}\leq P}\;\;\frac{\sigma_{1}^{2}+D}{\sigma_{1}^{2}}\cdot \frac{D+\sigma_{w}^{2}{\left(\gamma +1\right)}^{2}+\sigma_{1}^{2}+P_{A}{\left(\alpha +1\right)}^{2}}{D+\sigma_{w}^{2}{\left(\gamma +1\right)}^{2}+\sigma_{1}^{2}}\right). \end{array}$$

**Proof.**  Here note that Equation (9) is also the capacity of the action-dependent channel with noncausal state at the transmitter, and the capacity formula of its Gaussian case is be given in [13] by substituting Equations (26) and (25) into Equation (9) and maximizing the parameters δ and β. Now, directly using the Gaussian capacity formula in [13], we have Equation (37). The proof is completed. ☐

Finally, to show the feedback gain, we also provide a lower bound $C_{sag}^{in}$ on the secrecy capacity $C_{sag}$ of the action-dependent dirty paper wiretap channel (see Theorem 6).

**Theorem** **6.** *$C_{sag}\geq C_{sag}^{in}$, where*

(38) $$\begin{array}{c} C_{sag}^{in}=\max_{\alpha ,\gamma ,\delta ,\beta }\min\{\frac{1}{2}\log\left(\frac{E\left(Y^{2}\right)E\left(U^{2}\right)}{E\left(Y^{2}\right)E\left(U^{2}\right)-{\left(E\left(UY\right)\right)}^{2}}\right)-\frac{1}{2}\log\left(\frac{{\left(\gamma \delta +\beta \right)}^{2}\sigma_{w}^{2}+\delta^{2}D}{\delta^{2}D}\right),\\ \frac{1}{2}\log\left(\frac{E\left(Y^{2}\right)E\left(U^{2}\right)}{E\left(Y^{2}\right)E\left(U^{2}\right)-{\left(E\left(UY\right)\right)}^{2}}\right)-\frac{1}{2}\log\left(\frac{E\left(Z^{2}\right)E\left(U^{2}\right)}{E\left(Z^{2}\right)E\left(U^{2}\right)-{\left(E\left(UZ\right)\right)}^{2}}\right)\}, \end{array}$$

**Proof.**  In [20], a lower bound $C_{sa}^{in}$ on the secrecy capacity $C_{sa}$ of the discrete memoryless action-dependent wiretap channel with noncausal state at the transmitter is provided, and it is given by

(39) $$\begin{array}{c} C_{sa}^{in}=\max\min\left\{I\left(U;Y\right)-I\left(U;S|A\right),I\left(U;Y\right)-I\left(U;Z\right),H\left(A|Z\right)\right\}. \end{array}$$

Here, note that the term H(A|Z) in Equation (39) holds due to the assumption that the action encoder is a deterministic function of the transmitted message. Specifically, once the eavesdropper obtains the action sequence $A^{N}$, he knows the transmitted message, hence the achievable secrecy rate cannot exceed the eavesdropper’s uncertainty about $A^{N}$, i.e., H(A|Z).

In this paper, we use a stochastic action encoder instead of the deterministic one in [20], which indicates that, even if the eavesdropper obtains $A^{N}$, he does not know the transmitted message due to the randomness assumption of the action encoder. Hence. the term H(A|Z) no longer holds in this paper, i.e., for the action-dependent wiretap channel with noncausal state at the transmitter and stochastic action encoder, a lower bound $C_{sa}^{in*}$ is given by

(40) $$\begin{array}{c} C_{sa}^{in*}=\max\min\left\{I\left(U;Y\right)-I\left(U;S|A\right),I\left(U;Y\right)-I\left(U;Z\right)\right\}. \end{array}$$

Finally, substituting Equations (26) and (25) into Equations (40), Equation (38) is obtained. The proof is completed. ☐

**Figure 5:**
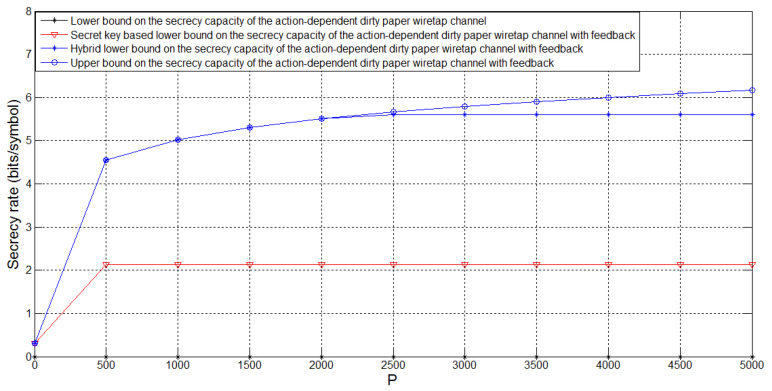
Comparison of the bounds on $C_{sag}^{f}$ for $P_{A}=1$, $\sigma_{w}^{2}=1$, $\sigma_{1}^{2}=1$, $\sigma_{2}^{2}=0.1$, t=0.9 and *P* taking values in [0,5000].

Figure 5 depicts the bounds on $C_{sag}^{f}$ and the lower bound $C_{sag}^{in}$ on the secrecy capacity of the action-dependent dirty paper wiretap channel for $\sigma_{w}^{2}=P_{A}=1$, $\sigma_{1}^{2}=1$, $\sigma_{2}^{2}=0.1$, t=0.9 and several values of *P*. For this case, we see that there is no positive achievable secrecy rate $C_{sag}^{in}$ of the action-dependent dirty paper wiretap channel, and feedback enhances $C_{sag}^{in}$. Moreover, we see that the hybrid feedback scheme performs better than the secret key based feedback scheme, and there exists a gap between the lower and upper bounds on $C_{sag}^{f}$ when *P* is sufficiently large.

**Figure 6:**
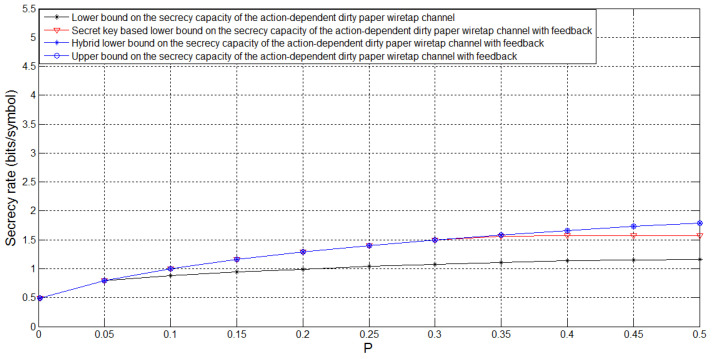
Comparison of the bounds on $C_{sag}^{f}$ for $P_{A}=1$, $\sigma_{w}^{2}=1$, $\sigma_{1}^{2}=0.1$, $\sigma_{2}^{2}=0.1$, t=0.6 and *P* taking values in [0,0.5].

**Figure 7:**
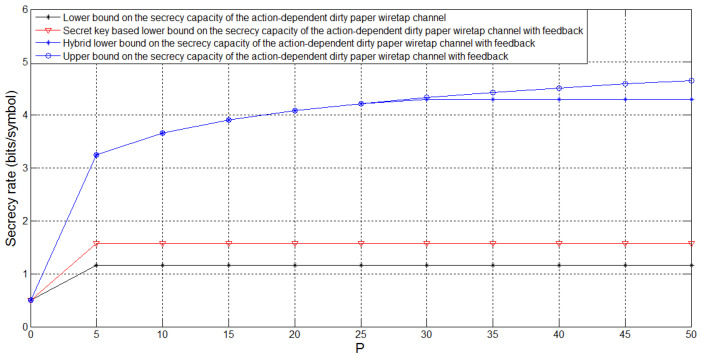
Comparison of the bounds on $C_{sag}^{f}$ for $P_{A}=1$, $\sigma_{w}^{2}=1$, $\sigma_{1}^{2}=0.1$, $\sigma_{2}^{2}=0.1$, t=0.6 and *P* taking values in [0,50].

Figure 6 depicts the bounds on $C_{sag}^{f}$ and the lower bound $C_{sag}^{in}$ on the secrecy capacity of the action-dependent dirty paper wiretap channel for $\sigma_{w}^{2}=P_{A}=1$, $\sigma_{1}^{2}=0.1$, $\sigma_{2}^{2}=0.1$, t=0.6 and *P* taking values in [0,0.5]. For this case, we see that feedback enhances $C_{sag}^{in}$, and the hybrid feedback scheme performs better than the secret key based feedback scheme. Moreover, we see that, when *P* is small, the hybrid feedback scheme is optimal, i.e., its corresponding lower bound meets the upper bound, which implies that the secrecy capacity $C_{sag}^{f}$ is determined for this case. Figure 7 is an extension of Figure 6 with *P* taking values in [0,50]. We see that, when *P* is sufficiently large, there exists a gap between the lower and upper bounds on $C_{sag}^{f}$, and eliminating this gap still has a long way to go.

## 5. Conclusions

In this paper, we propose two achievable secrecy rates for the action-dependent wiretap channel with noncausal state at the transmitter and feedback, where one rate is achieved by using the already existing secret key based feedback strategy, and the other is achieved by using a hybrid feedback strategy. From a Gaussian example (also called the action-dependent dirty paper wiretap channel with feedback), we show that both feedback strategies proposed in this paper enhance the achievable secrecy rate of the action-dependent dirty paper wiretap channel, and the hybrid feedback strategy performs better than the secret key based feedback strategy. Moreover, we show that the hybrid feedback strategy is optimal for some special cases.

## Abbreviations

The following abbreviations are used in this manuscript:

MDPI	Multidisciplinary Digital Publishing Institute
DOAJ	Directory of open access journals
TLA	Three letter acronym
LD	linear dichroism
